# *CSPG4P12* polymorphism served as a susceptibility marker for esophageal cancer in Chinese population

**DOI:** 10.1186/s12885-024-12475-4

**Published:** 2024-06-14

**Authors:** Hongxue Xu, Zhenbang Yang, Wenqian Hu, Xianlei Zhou, Zhi Zhang, Xuemei Zhang

**Affiliations:** 1 School of Public Health, North China University of Science and Technology, Tangshan, China; 2 College of Life Sciences, North China University of Science and Technology, Tangshan, China; 3 Hebei Key Laboratory of Occupational Health and Safety for Coal Industry, North China University of Science and Technology, Tangshan, China; 4 Affiliated Tangshan Gongren Hospital , North China University of Science and Technology, Tangshan, China

**Keywords:** *CSPG4P12*, Pseudogene, Esophageal cancer, Single nucleotide polymorphism, Genetic susceptibility

## Abstract

**Background:**

Chondroitin sulfate proteoglycan 4 pseudogene 12 (*CSPG4P12*) has been implicated in the pathogenesis of various cancers. This study aimed to evaluate the association of the *CSPG4P12* polymorphism with esophageal squamous cell carcinoma (ESCA) risk and to explore the biological impact of *CSPG4P12* expression on ESCA cell behavior.

**Methods:**

A case-control study was conducted involving 480 ESCA patients and 480 healthy controls to assess the association between the rs8040855 polymorphism and ESCA risk. The *CSPG4P12* rs8040855 genotype was identified using the TaqMan-MGB probe method. Logistic regression model was used to evaluate the association of *CSPG4P12* SNP with the risk of ESCA by calculating the odds ratios (*OR*) and 95% confidence intervals (95%*CI* ). The effects of *CSPG4P12* overexpression on cell proliferation, migration, and invasion were examined in ESCA cell lines. Co-expressed genes were identified via the CBioportal database, with pathway enrichment analyzed using SangerBox. The binding score of *CSPG4P12* to P53 was calculated using RNA protein interaction prediction (RPISeq). Additionally, Western Blot analysis was performed to investigate the impact of *CSPG4P12* overexpression on the P53/PI3K/AKT signaling pathway.

**Results:**

The presence of at least one rs8040855 G allele was associated with a reduced susceptibility to ESCA compared to the CC genotype (*OR* = 0.51, 95%*CI* = 0.28–0.93, *P* = 0.03). Stratification analysis revealed that the *CSPG4P12* rs8040855 C allele significantly decreased the risk of ESCA among younger individuals (≤ 57 years) and non-drinkers (*OR* = 0.31, 95%*CI* = 0.12–0.77, *P* = 0.01; *OR* = 0.42, 95%*CI*=0.20–0.87, *P* = 0.02, respectively). *CSPG4P12* expression was found to be downregulated in ESCA tissues compared to adjacent normal tissues. Overexpression of *CSPG4P12* in ESCA cells inhibited their proliferation, migration, and invasion capabilities. Furthermore, Western Blot analysis indicated that *CSPG4P12* overexpression led to a reduction in PI3K and p-AKT protein expression levels. P53 silencing rescues the inhibitory effect of *CSPG4P12* on p-AKT.

**Conclusion:**

The *CSPG4P12* rs8040855 variant is associated with reduced ESCA risk and the overexpression of *CSPG4P12* inhibited the migration and invasion of ESCA cells by P53/PI3K/AKT pathway. These findings suggest that *CSPG4P12* may serve as a novel biomarker for ESCA susceptibility and a potential target for therapeutic intervention.

**Supplementary Information:**

The online version contains supplementary material available at 10.1186/s12885-024-12475-4.

## Introduction

Esophageal cancer is the 6th most common cause of cancer-related deaths worldwide [1]. It is estimated that in 2022 there will be 346,633 new cases of esophageal cancer and 323,600 deaths in China, making it the fourth leading cause of cancer deaths [2]. Environmental factors, such as smoking, alcohol consumption and low intake of fresh fruits, have been found to be risk factors for esophageal cancer [3]. In addition to environmental factors, epidemiological and pathogenetic studies have confirmed that genetic variants also play an important role in the development of esophageal cancer [4]. In recent years, many genetic variants that affect the risk of developing esophageal cancer have been identified in genome-wide associated studies and candidate gene associated studies [5–8].

Most pseudogenes are long non-coding RNA(LncRNA), which are highly similar to parent genes but does not have protein-coding functions. Studied have found that pseudogene is involved in variety of physiological and biochemical processes, such as chromatin dynamics, gene expression, cell growth and their regulation [9]. During tumorigenesis, LncRNAs can regulate important cell signaling pathways such as the p53 pathway, at transcriptional and post-transcriptional level [10]. .

Single nucleotide polymorphism (SNP) is the most common variation in the human genome, accounting for more than 90% [11, 12]. Some genetic variants can affect the biological function of LncRNA by regulating its expression and contribute to the risk and prognosis of specific cancer [13–15]. For example, genetic variant, rs10505477 in LncRNA *CASC8* was associated with the risk of lung cancer and could predict the response of lung cancer patients to platinum-based drug therapy [16].

Chondroitin sulfate proteoglycan 4 (*CSPG4*) is involved in the progression of many cancers, such as undifferentiated thyroid cancer, squamous cell carcinoma of the head and neck, and basal breast cancer [17, 18]. *CSPG4* pseudogene 12 (*CSPG4P12*) is a LncRNA derived from a pseudogene, which is highly homologous to its parent gene *CSPG4* [19]. Our research has previously shown that *CSPG4P12* plays a role in inhibiting proliferation and metastasis of lung cancer cells while promoting apoptosis [20]. This discovery prompted us to search for regulatory variants within *CSPG4P12* that could affect its expression levels. Among these, the rs8040855 variant emerged as a significant regulator of *CSPG4P12* expression. Given the established impact of genetic variants on gene expression and the potential for such variations to connect single nucleotide polymorphisms (SNPs) with their target genes or transcripts [21–23], we chose to focus on rs8040855 in our study. To further explore the biological relevance of this finding, we investigated the expression levels of the *CSPG4P12* gene in esophageal cancer tissues and adjacent normal tissues, revealing differential expression patterns that supported the potential functional impact of the rs8040855 polymorphism. These findings enabled us to discover the link between rs8040855 and esophageal cancer. In this study, we aim to investigate the correlation between the *CSPG4P12* rs8040855 and the risk of esophageal cancer and to explore the effect of *CSPG4P12* in the development of esophageal cancer.

## Materials and methods

### Participants in the population study

This case-control study involved in 480 patients with esophageal cancer and 480 healthy controls. All cases consisted of pathologically confirmed primary esophageal cancer patients who had not been treated with radiotherapy or chemotherapy. Controls were randomly selected from the eligible healthy population and who underwent a health check-up at the hospital during the same period. These healthy individuals had no previous history of tumor or blood relationship with cases. All participants were recruited at North China University of Science and Technology Affiliated Tangshan Gongren and Affiliated Tangshan Renmin Hospital (Tangshan, China) [24]. General demographic data and behavioral data of the study population were collected by reviewing medical records and questionnaire methods [7]. This study was approved by the Institutional Review Board of North China University of Science and Technology (No.2,022,027). All participants signed a written informed consent form.

### DNA extraction and *CSPG4P12* rs8040855 genotype determination

DNA from the peripheral blood of all participants was extracted by using the blood extraction kit (TIANGEN Biotechnology, Beijing, China). All subjects were genotyped by using TaqMan-MGB probe. Primers were listed as follows: rs8040855F: 5’-AAGGCTTCCTTGTGACACAGAAG-3’, rs8040855R: 5’-AGACCAAGGTTTGTATGCCCAC-3’, rs8040855-C Probe: FAM-CACATCTAGTTCTTTCTT-MGB, rs8040855-G Probe: VIC-CACATTACTCTTTCTT-MGB. PCR reaction was carried out in a 5 µl mixture containing 0.2 µl (2 µmol/L) of each probe, 0.15 µl (10 µmol/L) of each primer, and 1 µl (0.1–20 ng) of genomic DNA. 1×PCR mix (TaqMan Universal Master Mix II, ABI, USA). The PCR procedure consisted of an initial melting step of 2 min at 50 °C, 10 min at 95 °C, followed by 50 cycles of 15 s at 95 °C and then 1 min at 58 °C. ABI SDS 2.4 software was used to judge genotypes. Genotyping results were repeated with 10% of the samples. Blank controls were set in each plate to avoid erroneous results due to contamination.

### The construction of *CSPG4P12* overexpression vector

The *CSPG4P12-*pUC57 (7.54 kb) plasmid was Synthesized from Changzhou Ruibo Biotechnology (Jiangsu, China). In brief, *CSPG4P12* (ENST00000558282.5; *CSPG4P12*-201; 4853 bp) was amplified using KOD FX (cat. no. KFX-101; TOYOBO, Osaka, Japan), and the following primers (SinoGenoMax, Beijing, China): 5’-CTAGTCTAGACACCTGGGCACCAACCTC-3’ (with XbaI cutting site) and 5’-ACGCGTCGACATAGAAAACAGCCCCAACCAG-3’ (with SaII cutting site). The thermocycling conditions: Pre-denaturation at 95˚C for 3 min; followed by 25 cycles at 95˚C for 25 s, at 60˚C for 20 s, and 72˚C for 40 s; final extension at 72˚C for 1 min. The PCR product was recombined into the pUC57 vector to generate the *CSPG4P12* overexpression plasmid (*CSPG4P12-*pUC57), which was verified by Sanger sequencing.

### Cell culture and plasmid treatment

Esophageal cancer cells (KYSE-30 and TE-10) (Procell Life Science & Technology, Wuhan, China) were cultured in RPMI 1640 medium (Thermo Fisher Scientific, Waltham, MA, USA) supplemented with 10% FBS (Zhejiang Tianhang Biotechnology, Zhejiang, China) at 37˚C with 5% CO_2_. The cells were regularly tested for mycoplasma contamination. Cells were seeded into six‑well plates at a density of 1 × 10^6^ cells/well. When cells reach 80% confluency, *CSPG4P12*‑pUC57 or empty plasmid (pUC57) were transfected into KYSE-30 and TE-10 cells using Lipofectamine^®^ 2000 (Thermo Fisher Scientific, Waltham, MA, USA) for 5 h at 37˚C. Cells were then harvested for further analysis after 24 h. The transfected cells continued to be cultured in with or without 20 µM/L PFT-α (MedChemExpress, New Jersey, USA) for 24 h.

### Total RNA extraction and the detection of *CSPG4P12* mRNA

To detect *CSPG4P12* RNA expression, total RNA was extracted from the cells using the TRIzol^®^ reagent (Thermo Fisher Scientific, Waltham, MA, USA) according to the manufacturer’s protocol. The RNA was then treated with DNase I (Thermo Fisher Scientific, Waltham, MA, USA) to remove any residual genomic DNA contamination. The DNase-treated RNA was then reverse-transcribed into cDNA using the RevertAid First Strand cDNA Synthesis kit (Thermo Fisher Scientific, Waltham, MA, USA). The cDNA was used as a template for real-time fluorescence quantitative PCR to measure *CSPG4P12* expression. For SYBR Green-based qPCR, the 10µl reaction included 100 ng of cDNA, 5µl of 2×Power SYBR‑Green PCR Master Mix (Thermo Fisher Scientific, Waltham, MA, USA) in a 7900HT Fast Real‑Time PCR System (Thermo Fisher Scientific, Waltham, MA, USA) with GAPDH as the internal reference. The primer pairs for RT‑qPCR are *CSPG4P12* F/*CSPG4P12*-R (5’‑ATGGACCAGTACCCCACACG‑3’/ 5’‑CCCTGCCTCTAGCCATTGAC‑3’) and *GAPDH*- F/*GAPDH*-R (5’‑CTGGGCTACACTGAGGACC‑3’/5’‑AAGTGGTCGTTGAGGGCAATG‑3’). The thermocycling conditions were as follows: pre‑denaturation at 95˚C for 2 min; followed by 40 cycles at 95˚C for 15 s and at 59˚C for 1 min; final extension at 72˚C for 10 min. The relative expression levels of *CSPG4P12* were calculated using the 2^‑∆∆Ct^ method [25].

### Cell biology phenotypes

CCK-8 assay was used to detect cell viability. A total of 1 × 10^4^ transfected cells/well were seeded into 96-well plates. Incubated cells at 37˚C for 24, 48 and 72 h and then let stand at 37˚C for 1 h after adding 10 µl CCK-8 reagent (Dojindo, Kyushu, Japan). The absorbance at 450 nm was measured using the Infinite M200 PRO instrument (Tecan, Männedorf, Switzerland). Cell migration and invasion were determined by Transwell assay. For invasion assay, the upper chamber of Transwell was precoated with Matrigel (Corning, NY, USA) for 5 hours at 37 °C. Then, 1.5 × 10^5^ cells were seeded in 200µL RPMI 1640 medium into an 8 μm pore size upper chamber (JET BIOFIL, Guangzhou, China) for 48 h. Matrigel (8–11 mg/ml) was mixed with RPMI 1640 medium in a 1:8 ratio, and the final concentration of Matrigel used in the invasion assay was 0.9 to 1.2 mg/ml. For migration assay, cells were directed seeded in the upper chamber without Matrigel. The lower chamber is filled with 600 µl RPMI 1640 medium supplemented with 20% FBS.

### Bioinformatics analysis

Online ncRNA-eQTL program (http://ibi.hzau.edu.cn/ncRNA-eQTL/index.php) was used to analyze the effect of rs8040855 on the expression level of *CSPG4P12*. All biological analysis data were downloaded from UCSC XENA (https://xenabrowser.net/datapages/). DESeq2 was used to analyze differences in *CSPG4P12* expression between esophageal cancer tissues and adjacent normal tissues, setting |log2(FC)|>1 & *P* < 0.05 as the thresholds for difference analysis. Cox regression analysis was used to analyze the effect of *CSPG4P12* expression level on the prognosis of patients. R survival [v 3.3.1] and ggplot2 [v 3.3.6] were used for this analysis. The cBioportal database (https://www.cbioportal.org) was used to screen for *CSPG4P12* co-expressed genes. The *q*-Value < 0.05 was used as a screening condition. Sangerbox (http://vip.sangerbox.com/) online website was used for enrichment analysis of the signal pathways acted by *CSPG4P12* co-expressed genes. The binding score of *CSPG4P12* to P53 was calculated using RNA protein interaction prediction (RPISeq) (http://pridb.gdcb.iastate.edu/RPISeq/).

### Western blotting analysis

The esophageal cancer cells were lysed with radioimmunoprecipitation assay (RIPA) buffer (Thermo Fisher Scientific, Waltham, MA, USA). The Pierce BCA Protein Assay Kit (Solarbio, Beijing, China) was used to detect protein concentrations. The samples underwent separation through 10% sodium dodecyl sulfate-polyacrylamide gel electrophoresis (SDS-PAGE), before being transferred to a polyvinylidene difluoride (PVDF) membrane (Millipore, Billerica, MA, USA) for additional analysis. Following a 2-hour blocking step at room temperature with 5% skimmed milk, the membrane was exposed to the primary antibody overnight at 4 °C. Subsequently, the membrane was washed and incubated with a horseradish peroxidase (HRP)-conjugated secondary antibody for 2 h at room temperature. The specific protein was subsequently visualized using enhanced chemiluminescence (ECL) luminescence reagents (Amersham, Slough, Buckinghamshire, UK). Due to the constraints of our experimental setup, some blots were cut prior to hybridization with antibodies to facilitate simultaneous probing for multiple targets. This step was necessary to ensure the efficient use of samples and reagents. Consequently, full-length images of some blots are not available. Where full-length images are not available, we have included the original blots with visible membrane edges in the Supplementary Information file. β-Actin was applied as a reference control. The following primary antibodies were used: anti − PI3K (1:1,000 dilution; ab191606; Abcam, Cambridge, UK), anti −AKT (1:10,000 dilution; ab179463; Abcam), anti − p-AKT (1:1,000 dilution; ab192623; Abcam), anti − NF-κB p65 (1:5,000 dilution; ab32536; Abcam), anti − p- NF-κB p65 (1:1,000 dilution; ab76302; Abcam), and P53 Polyclonal Antibody (1:2,000 dilution; 10442-1-AP; Proteintech).

### Statistical analysis

SPSS 23.0 software was used for statistical analysis. Chi-square tests were used to compare the distributions of categorical variables. Odds ratios (*OR*), and 95% confidence intervals (*CI*) were calculated to evaluate the association of rs8040855 genotype with the susceptibility to esophageal cancer using unconditional logistic regression. All statistical tests were two-sided t-tests. *P* < 0.05 is considered statistically significant.

## Results

### Differential expression of *CSPG4P12* in ESCA and SNP selection

**Fig. 1:**
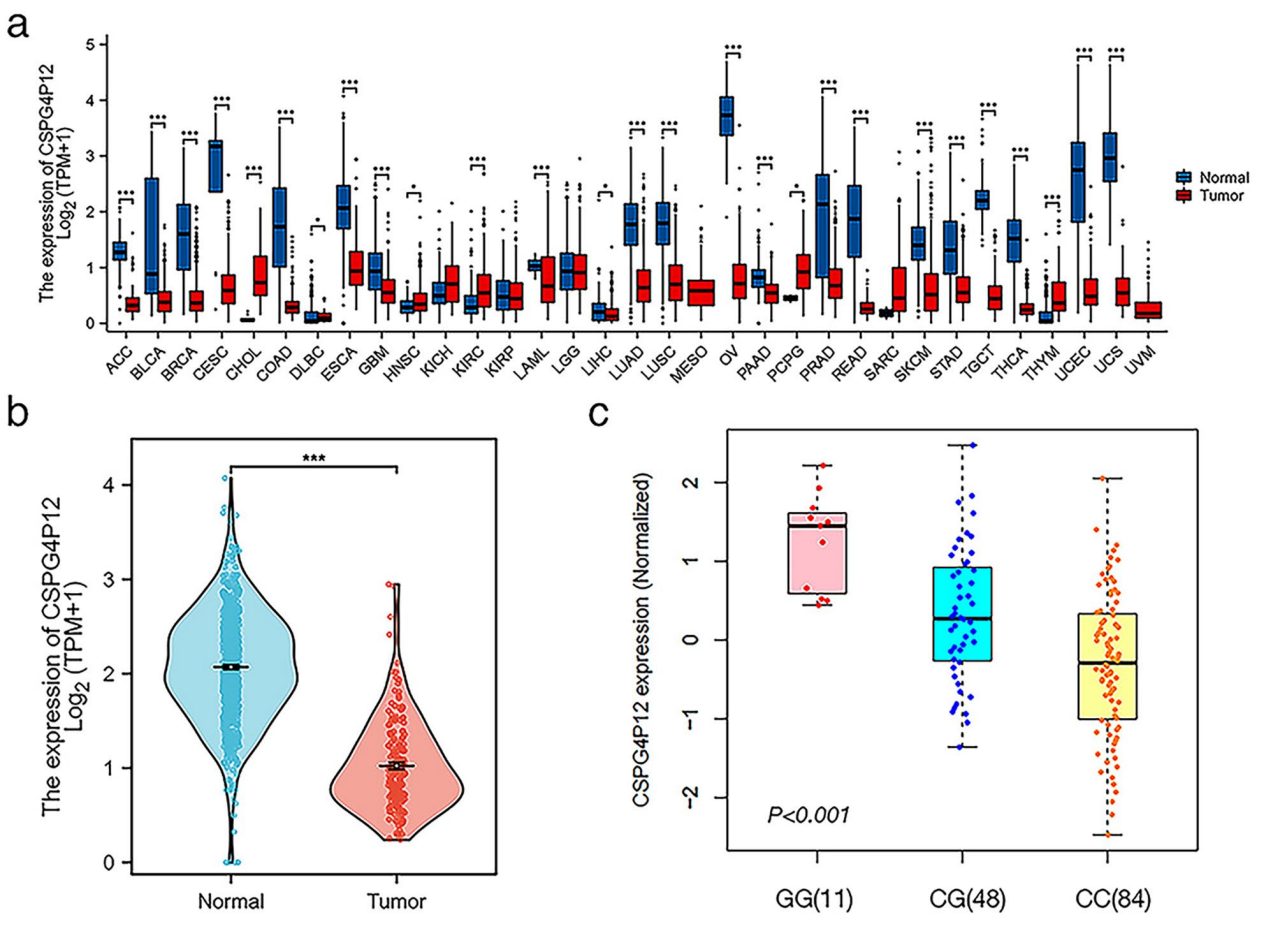
Expression of *CSPG4P12* in ESCA (**a**) Pan-cancer analysis of *CSPG4P12* differential expression. (**b**) Expression level of *CSPG4P12* in ESCA tumor tissues. (**c**) Effect of *CSPG4P12* rs8040855 genotyping on the expression level of *CSPG4P12*

Bioinformatic analysis revealed that the pseudogene *CSPG4P12* is poorly expressed in many types of cancer tissue, including esophageal cancer (Fig. 1a and b). Our ncRNA-eQTL result showed that rs8040855 CG or GG genotype significantly increases the expression level of *CSPG4P12* compared to the CC genotype. (Fig. 1c).

### Correlation analysis between *CSPG4P12* rs8040855 polymorphism and the risk of ESCA

The general demographic information of the subjects is listed in Table 1. There was no significant difference in gender and age composition between the cases and controls. More people smoked cigarette (58.3%) and drank alcohol (30.8%) in case group (32.5%, 21.7%). In normal controls, the frequency of *CSPG4P12* rs8040855 CC and CG genotype was 93.1% and 6.9%, respectively, which was in line with Hardy-Weinberg equilibrium (χ^2^ = 0.61, *P* = 0.44). It is worth noting that we did not detect the GG genotype in normal controls. The *CSPG4P12* rs8040855 affected the risk of susceptibility to esophageal cancer (Table 2). The carriers with rs8040855 CG or GG genotype had reduced susceptibility to esophageal cancer compared to CC genotype with *OR* (95%*CI*) of 0.51(0.28–0.93) (*P* = 0.03).

**Table 1:** Distributions of select characteristics in cases and control subjects

Variables	Case(*n* = 480)			Controls(*n* = 480)		*P* value
	*N*	(%)		*N*	(%)	
Gender						0.70
Male	378	78.7		373	77.7	
Female	102	21.3		107	22.3	
Age						0.56
≤ 57	213	44.4		222	46.3	
> 57	267	55.6		258	53.7	
Smoke						*< 0.01*
No	200	41.7		324	67.5	
Yes	280	58.3		156	32.5	
Alcohol						*< 0.01*
No	332	69.2		376	78.3	
Yes	148	30.8		104	21.7	

**Table 2:** Gene polymorphism of *CSPG4P12* and their association with ESCA

CSPG4P12 genotypes	Case(*n* = 480)		Controls(*n* = 480)		*OR* (95%*CI*)^a^	*P* value
	*N*	(%)	*N*	(%)		
CC	462	96.3	447	93.1	1.00(Ref)	
CG + GG^b^	18	3.7	33	6.9	0.51(0.28–0.93)	*0.03*

^a^ Data were analyzed by unconditional logistic regression and adjusted for sex, age, smoking status and drinking status

^b^ The number of GG genotypes was less than 10 and was combined with CG genotypes for analysis

### Stratification analysis of the association of *CSPG4P12* variant with the risk of esophageal cancer

The stratification analysis results were list in Table 3. The carriers with CG or GG genotype had a lower risk of esophageal cancer compared to CC genotype in the younger age group (age ≤ 57) (*OR* = 0.31, *95%CI* = 0.12–0.77, *P* = 0.01), but not in the elder age group (age > 57). The individuals with CG or GG carriers had a lower risk of esophageal cancer compared to CC genotypes among non-drinkers, (*OR* = 0.42, *95%CI*: 0.20–0.87, *P* = 0.02), but not among drinkers.

**Table 3:** Stratified analysis between *CSPG4P12* rs8040855 genotypes and ESCA risk

Variables	Genotypes (Cases/Controls)		CG + GG/CC model *OR* (95%*CI*)^a^	*P* value
	CC	CG + GG		
Gender				
Male	364/350	14/23	0.54(0.27–1.11)	0.09
Female	98/97	4/10	0.42(0.13–1.38)	0.15
Age				
≤ 57	206/203	7/19	0.31(0.12–0.77)	*0.01*
> 57	256/244	11/14	0.82(0.35–1.89)	0.64
Smoke				
No	193/302	7/22	0.46(0.19–1.10)	0.08
Yes	269/145	11/11	0.56(0.24–1.33)	0.19
Alcohol				
No	321/349	11/27	0.42(0.20–0.87)	*0.02*
Yes	141/98	7/6	0.81(0.25–2.56)	0.72

^a^ Data were analyzed by unconditional logistic regression and adjusted for sex, age, smoking status and drinking status

### Overexpression of *CSPG4P12* inhibits ESCA cell proliferation and migration-invasive ability

**Fig. 2:**
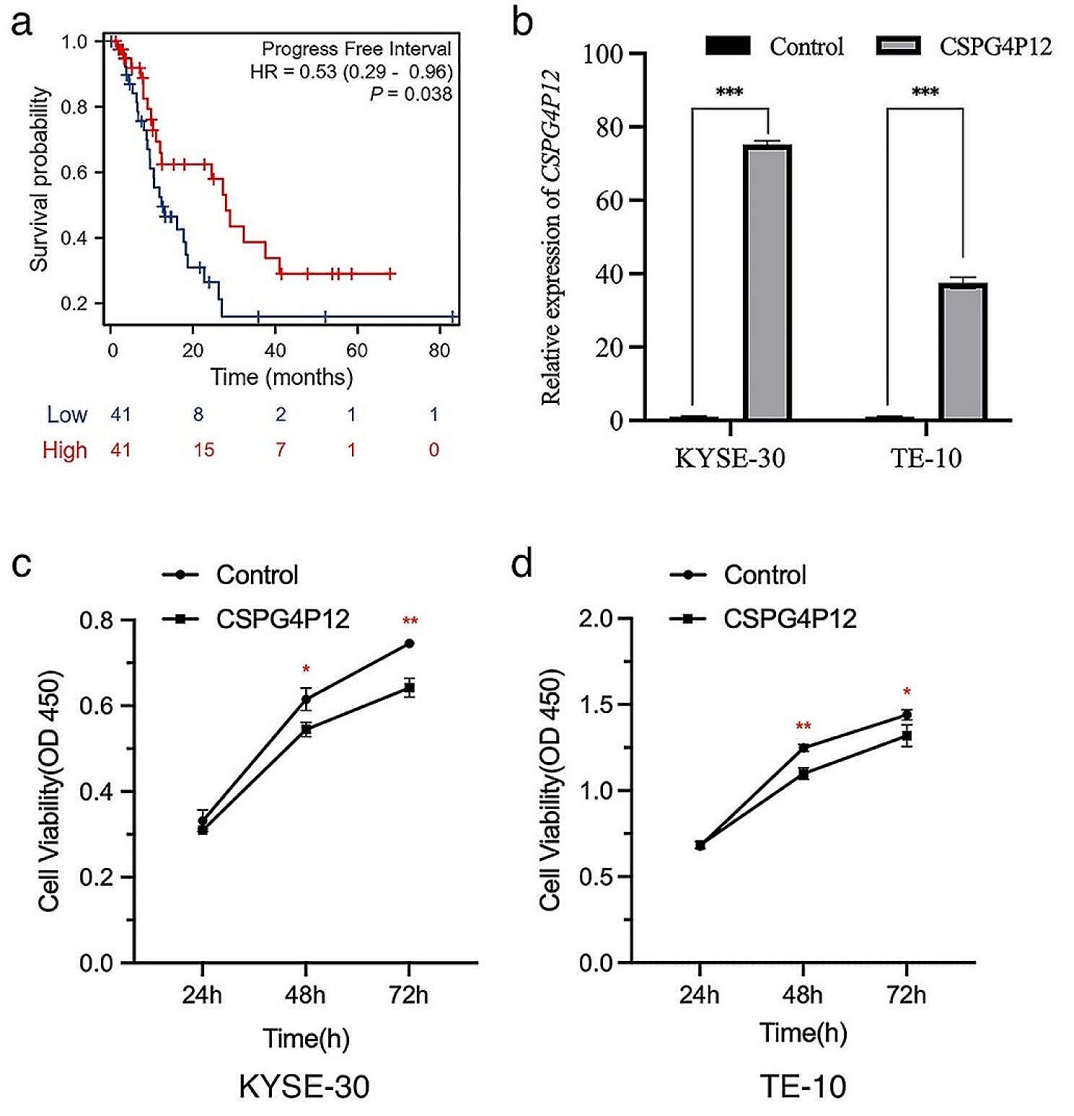
The effect of *CSPG4P12* on ESCA (**a**) Low expression of *CSPG4P12* is associated with poor prognosis of ESCA. (**b**) *CSPG4P12* was overexpressed in ESCA cells. (**c**, **d**) Overexpression of *CSPG4P12* inhibited the proliferation of ECSA cells

**Fig. 3:**
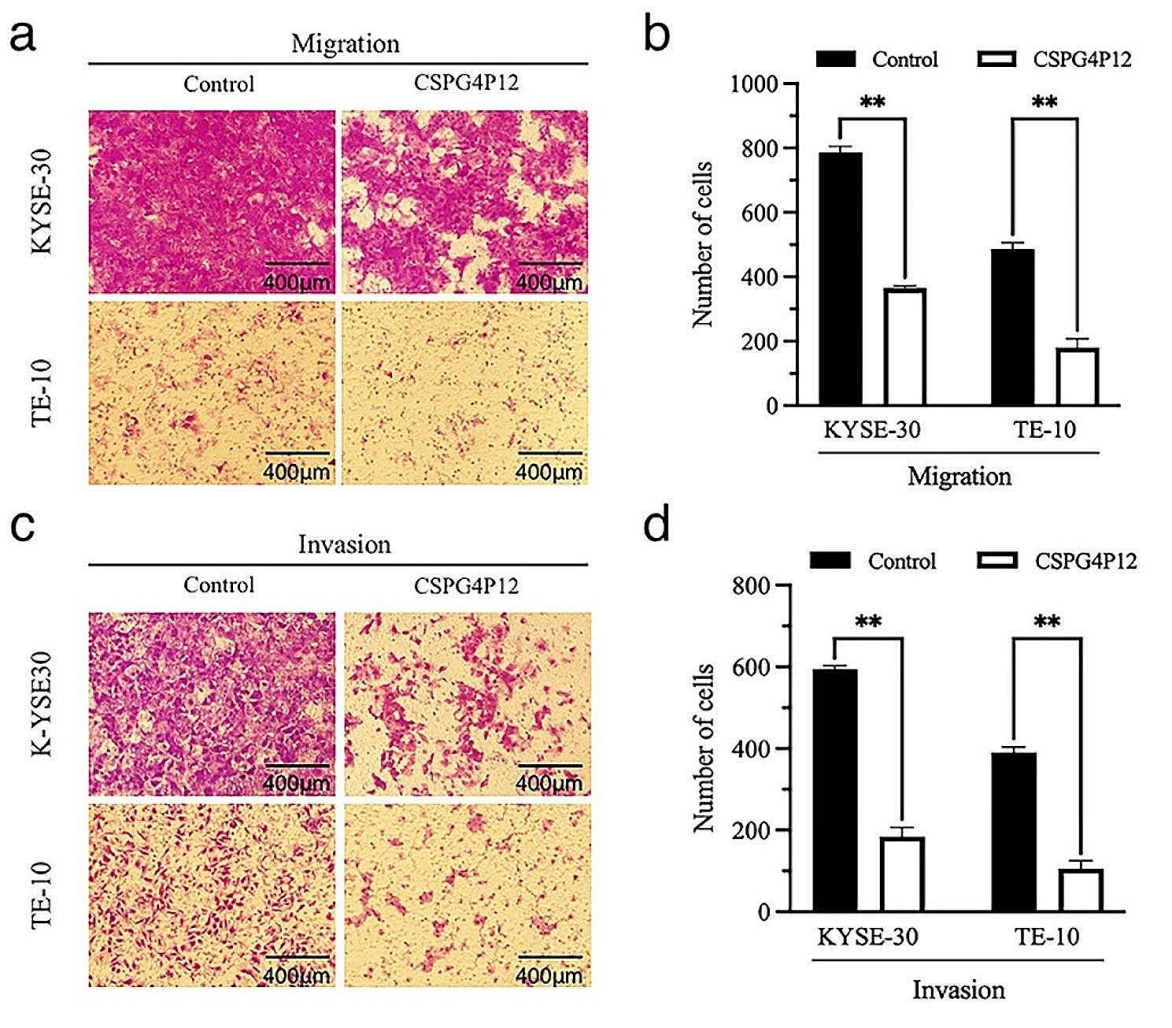
Effects of *CSPG4P12* overexpression on migratory and invasive abilities of ESCA cells (**a**) Detection of ESCA cell migration using Transwell assay (magnification, x100); (**b**) the results of which were quantified. (**c**) Detection of ESCA cell invasion using Transwell assay (magnification, x100); (d) the results of which were quantified

We found that the low expression of *CSPG4P12* was associated with the poor prognosis of esophageal cancer based on UCSC XENA data. (Fig. 2a). We then constructed *CSPG4P12* overexpression vector to explore its role in the development of esophageal cancer. *CSPG4P12* expression in KYSE-30 (73-fold) and TE-10 (37-fold) was elevated by 73- and 37-fold respectively, after transfected *CSPG4P12*-pUC57 overexpression plasmid. (Fig. 2b). CCK-8 assay showed that the overexpression of *CSPG4P12* significantly reduced the viability of KYSE-30 and TE-10 cells in a time dependent manner (*P* < 0.05) (Fig. 2c and d). Transwell assay indicated that *CSPG4P12* significantly decreased the migration and invasion ability of esophageal cancer cells (*P* < 0.01) (Fig. 3).

### Overexpression of *CSPG4P12* induces P53 inhibition of PI3K/AKT

**Fig. 4:**
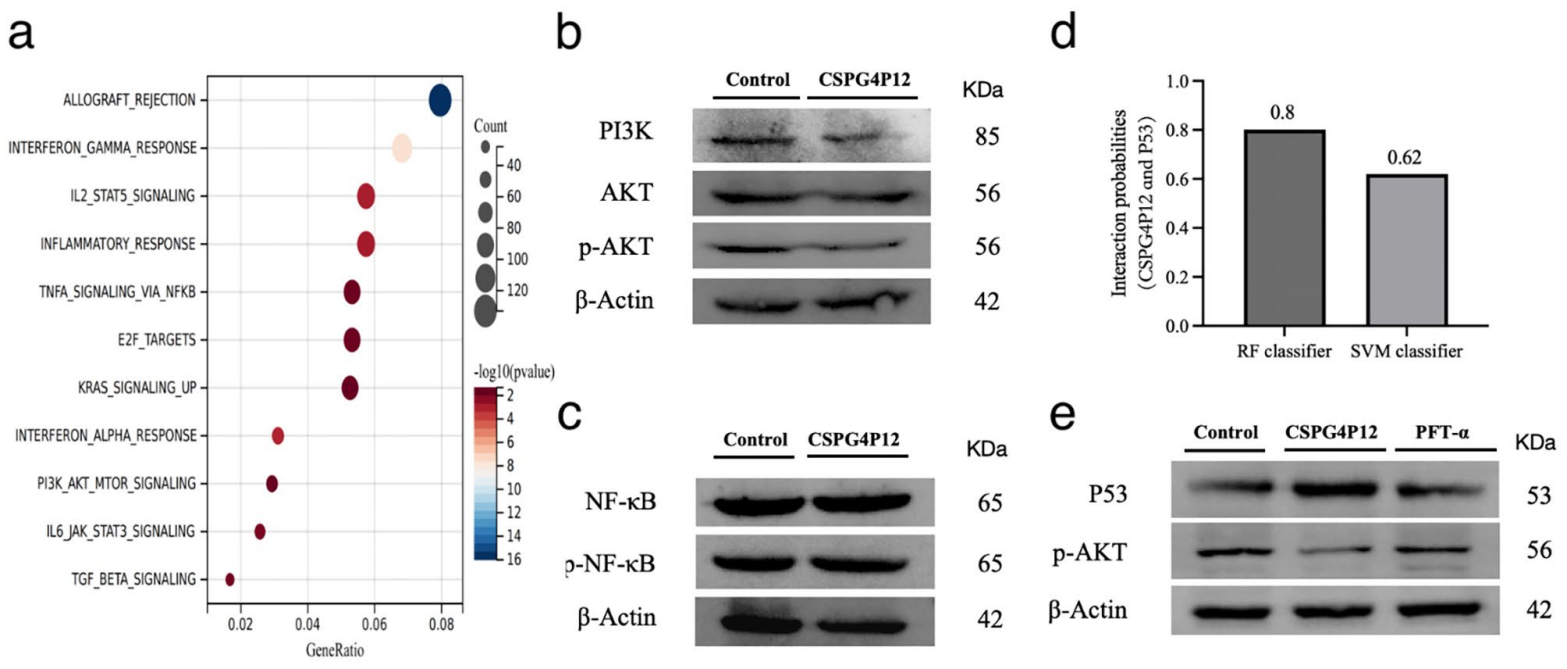
Effects of *CSPG4P12* overexpression on P53/PI3K/AKT. (**a**) Enrichment analysis of signal pathways for *CSPG4P12* co-expressed genes action; (**b**) Detection of PI3K/AKT protein expression using western blotting; (**c**) Detection of NF-κB protein expression using western blotting; (**d**) The binding probability of *CSPG4P12* and P53 was predicted by RPISeq database; (**e**) P53 silencing rescues the inhibitory effect of *CSPG4P12* on p-AKT

To further explore the mechanism of *CSPG4P12* in ESCA, we enriched the signal pathways in which *CSPG4P12* co-expressed genes acted. The results showed that *CSPG4P12* co-expressed genes mainly acted on signal pathways such as PI3K/AKT and NF-KB, which are closely related to tumors (Fig. 4a). The results of protein blotting experiments revealed that overexpression of *CSPG4P12* could reduce the levels of PI3K and p-AKT protein expression but did not affect the level of NF-κB protein expression (Fig. 4b and c). Further interaction analysis showed that *CSPG4P12* may bind to P53 at RF and SVM scores greater than 0.6. And our previous study found that overexpression of *CSPG4P12* significantly increased P53 expression (Fig. 4.d). We analyzed the effect of *CSPG4P12* on p-AKT by inhibiting P53. The results showed that silencing of P53 could effectively rescue the inhibitory effect of *CSPG4P12* on p-AKT (Fig. 4.e). These studies demonstrated that overexpression of *CSPG4P12* inhibited the migration and invasion ability of ESCA cells by inducing P53 to inhibit the PI3K/AKT signaling pathway.

## Discussion

Pseudogenes are involved in the process of esophageal cancer development [26, 27]. For example, *MALAT1* was found to regulate esophageal cancer growth by modifying the ATM-CHK2 pathway in esophageal cancer [28]. The knockdown of *MALAT1* in esophageal cancer can inhibit the migration and invasion ability of esophageal cancer cells. *CSPG4P12*, as a highly homologous pseudogene of *CSPG4*, most likely possesses a similar biological role to *CSPG4* which has been demonstrated to play an important role in tumor cell growth and metastasis [17].

In this study, our data showed that the pseudogene *CSPG4P12* is lowly expressed in esophageal cancer tissues compared to adjacent normal tissues. Similarly, our previous study found that *CSPG4P12* expression was downregulated in NSCLC tissues [20]. This study also provided evidence that *CSPG4P12* could inhibit the proliferation, migration, and invasion ability of esophageal cancer cells. The PI3K/AKT signal pathway is an intracellular signal transduction pathway that responds to extracellular signals to promote metabolism, proliferation, cell survival, growth and angiogenesis, and it is closely related to ESCA development [29, 30]. A study showed that *G3BP1* deficiency could inhibit the proliferation, migration and invasion of ESCA cells through the PI3K/AKT pathway [31]. In addition, it was found that knockdown of *DEAD-box 51* inhibited the tumor growth of ESCA through the PI3K/AKT pathway [32]. In addition, our finding showed a significant upregulation of the P53 protein upon overexpression of *CSPG4P12*. Although P53 is primarily known for its roles in cell cycle regulation and apoptosis, it can also report to negatively regulate the PI3K/AKT pathway [33]. This suggests that *CSPG4P12* inhibits the proliferation and migratory invasive ability of ESCA cells by suppressing the PI3K/AKT pathway through increased P53 levels.

It is pivotal to highlight the significant findings from our previous studies that demonstrate the profound impact of *CSPG4P12* overexpression on pivotal cellular pathways controlling cell cycle and apoptosis. Specifically, the overexpression of *CSPG4P12* markedly enhances the expression of P53, a critical tumor suppressor gene [20]. The upregulation of P53 is known to promote cell cycle arrest and apoptosis, thus acting as a fundamental checkpoint in preventing tumor progression [34]. Concurrently, *CSPG4P12* overexpression results in a significant reduction in the levels of Bcl-2 [20], an anti-apoptotic protein that typically contributes to cell survival and resistance to cell death mechanisms in cancer cells [35]. The decrease in Bcl-2 expression could lead to enhanced apoptotic activity within cells, further underscoring the potential of *CSPG4P12* as a modulator of cell fate decisions. These observations suggest that *CSPG4P12* may serve as a critical regulatory lncRNA in oncogenesis. In a hepatocellular carcinoma (HCC) study, researchers demonstrated that *CSPG4P12*, combining 5 other genes (*BX537318.1*, *TMEM147*, *AC015908.3*, *CEBPZOS*, and *SRD5A3*), served as the signature for prognostic evaluation of HCC [36]. All these studies revealed a potential biological role of *CSPG4P12* in the development of various cancers. In our investigation into the role of *CSPG4P12* lncRNA in esophageal cancer, we assessed its expression across various esophageal cancer cell lines. The analyses consistently revealed low expression levels of *CSPG4P12* across all tested lines. Given this uniform low expression, we opted not to pursue knockdown experiments to study potential effects on the P53/PI3K/AKT signaling pathway. However, the importance of understanding the functional roles of *CSPG4P12*, particularly its interaction with the P53/PI3K/AKT pathway, cannot be fully understated. This pathway is crucial for numerous cellular processes, including growth, proliferation, and survival, and is often dysregulated in cancer. For future research, it would be advantageous to utilize esophageal cancer cell lines with naturally higher levels of *CSPG4P12*.

In this study, we also discovered the effect of genetic variants in *CSPG4P12* gene on the susceptibility to esophageal cancer. To date, this is the first report on the association of *CSPG4P12* polymorphism with the risk of any cancer. There is also no Chinese population data related to this polymorphism in NCBI database. In this study, we found that rs8040855 C > G variant significantly reduced the risk of esophageal cancer. This is consistent with our expression analysis result which showed that the change of rs8040855C to G increased the expression of *CSPG4P12*. We also addressed the expression of *CSPG4P12* and its association with genetic polymorphisms in esophageal cancer using the online ncRNA-eQTL database due to the unavailability of direct tissue samples. As with any database derived from population studies, there are potential confounding factors such as age, gender, ethnicity, and environmental influences that might affect gene expression data but are not accounted for in eQTL analysis. Despite these limitations, utilizing ncRNA-eQTL data is a pragmatic interim solution that allows us to continue our research under current constraints. In our initial study design was primarily focused on establishing a genetic association between the *CSPG4P12* rs8040855 variant and esophageal cancer risk, along with its impact on gene expression levels. Expanding the study to include direct functional analysis of this variant in cell lines would require a significant shift in our research focus and resources, which was not feasible at this stage. Recognizing this limitation, we will investigate the functional role of the *CSPG4P12* rs8040855 variant in esophageal cancer in future research. These will be crucial for elucidating the molecular mechanisms by which the *CSPG4P12* rs8040855 variant contributes to esophageal cancer pathogenesis and could reveal novel targets for therapeutic intervention.

Our result also presented that *CSPG4P12* rs8040855 C > G genetic variant reduced the risk of esophageal cancer in non-drinkers, but not in drinkers. It is well known that alcohol consumption is a major risk factor for esophageal cancer [37, 38]. The ethanol in alcoholic beverages is metabolized into acetaldehyde, which has been classified as a human carcinogen by the International Agency for Research on Cancer [39]. In addition, the interaction between alcohol and genetic factors has been reported to have an effect on the risk of esophageal cancer [40]. In addition, genetic inheritance variations can reduce the risk of esophageal cancer in younger people. For example, it was found that the *POLR2E* rs3787016 C > T and *HULC* rs7763881 A > C genetic variants were factors in the reduced risk of esophageal cancer in the younger [41]. However, for this gene-environmental interaction result, a larger sample size are still needed.

## Conclusion

In summary, this study investigated the relationship between the polymorphisms and expression of *CSPG4P12* and ESCA risk based on a population-based case-control study design. The SNP rs8040855 genetic variation reduces the risk of ESCA. The rs8040855 C > G variant was related to the expression level of *CSPG4P12*. Moreover, the overexpression of *CSPG4P12* inhibited the migration and invasion of ESCA cells by P53/ PI3K/AKT. *CSPG4P12* may serve as a novel potential therapeutic target for ESCA. However, further studies in large populations and more functional investigations are warranted to validate the findings in this study.

### Electronic supplementary material

Below is the link to the electronic supplementary material.

Supplementary Material 1

Supplementary Material 2

Supplementary Material 3

Supplementary Material 4

## Data Availability

The datasets generated or analyzed during this study are included in this article.

## Abbreviations

ESCA: Esophageal cancer
LncRNA: Long non-coding RNA
SNP: Single nucleotide polymorphism
CSPG4: Chondroitin sulfate proteoglycan 4
CSPG4P12: CSPG4 pseudogene 12
OR: Odd ratio
CI: Confidence interval
CCK-8: Cell Counting Kit-8 Assay
RT-qPCR: Reverse Transcription- quantitative PCR
