# Supplementary figures and images for: CSPG4P12 polymorphism served as a susceptibility marker for esophageal cancer in Chinese population
Source: BMC Cancer. 2024 Jun 14;24:729. doi: 10.1186/s12885-024-12475-4 (PMC11177360; doi:10.1186/s12885-024-12475-4)

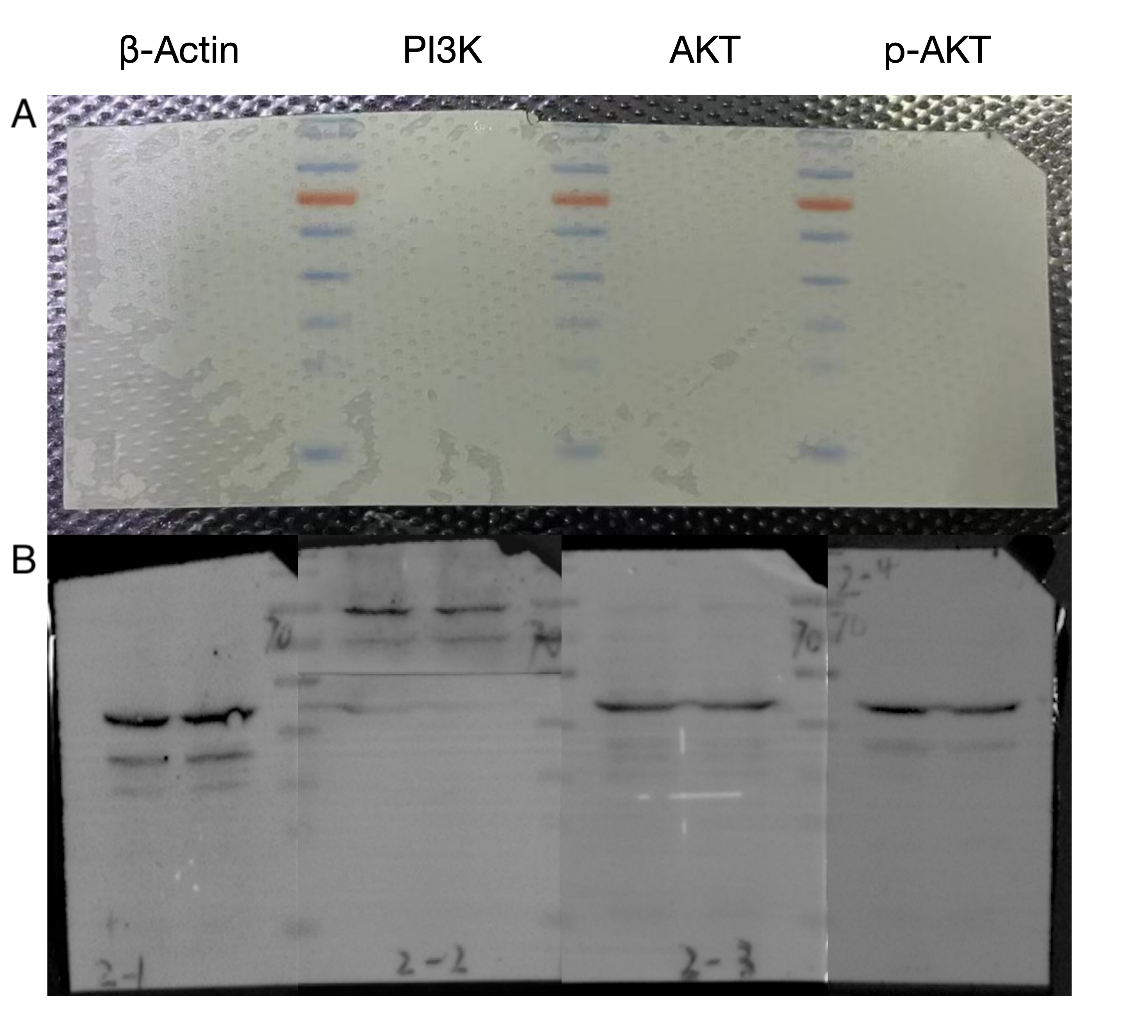

Supplement: Supplementary file 1 — Supplementary Material 1 [file 12885_2024_12475_MOESM1_ESM.png]

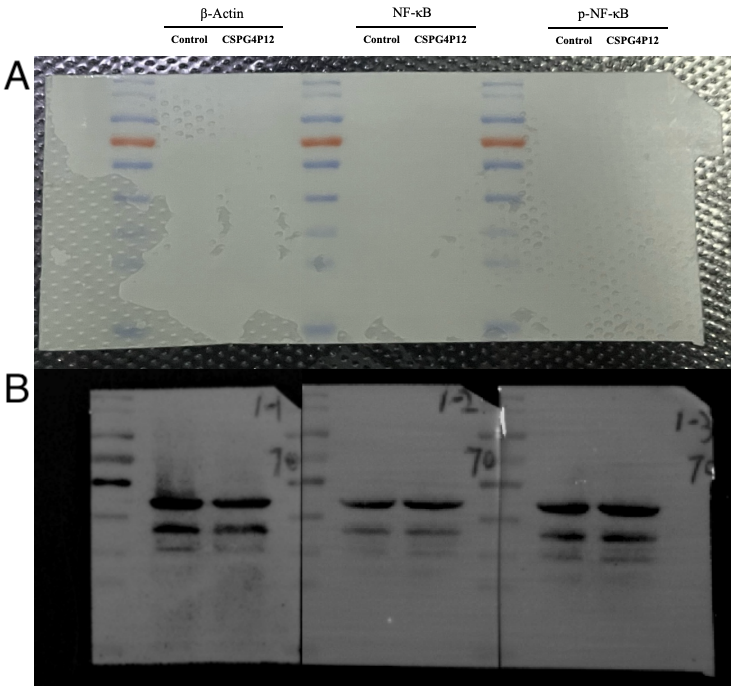

Supplement: Supplementary file 2 — Supplementary Material 2 [file 12885_2024_12475_MOESM2_ESM.png]

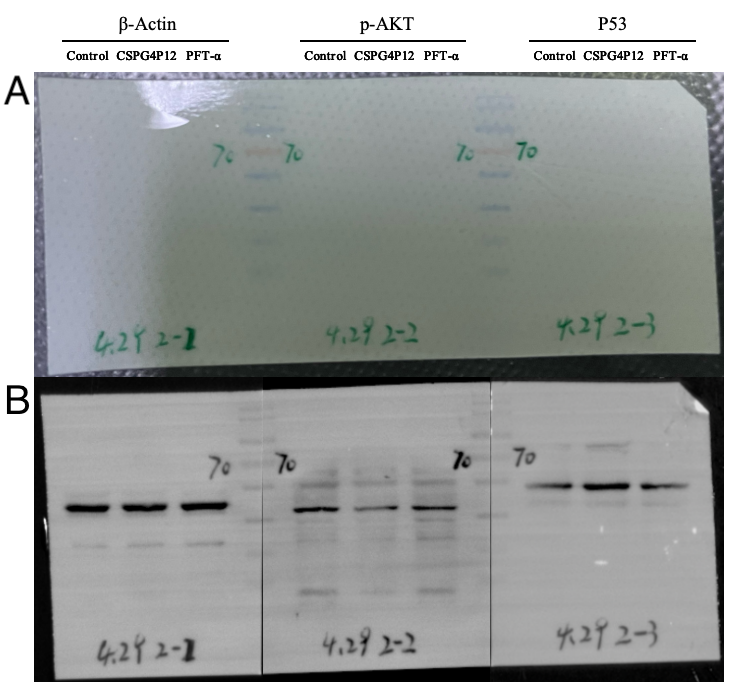

Supplement: Supplementary file 3 — Supplementary Material 3 [file 12885_2024_12475_MOESM3_ESM.png]
