# Supplementary material for: CSPG4P12 polymorphism served as a susceptibility marker for esophageal cancer in Chinese population
Source: BMC Cancer. 2024 Jun 14;24:729. doi: 10.1186/s12885-024-12475-4 (PMC11177360; doi:10.1186/s12885-024-12475-4)
Supplement: Supplementary file 4 — Supplementary Material 4 [file 12885_2024_12475_MOESM4_ESM.pdf]

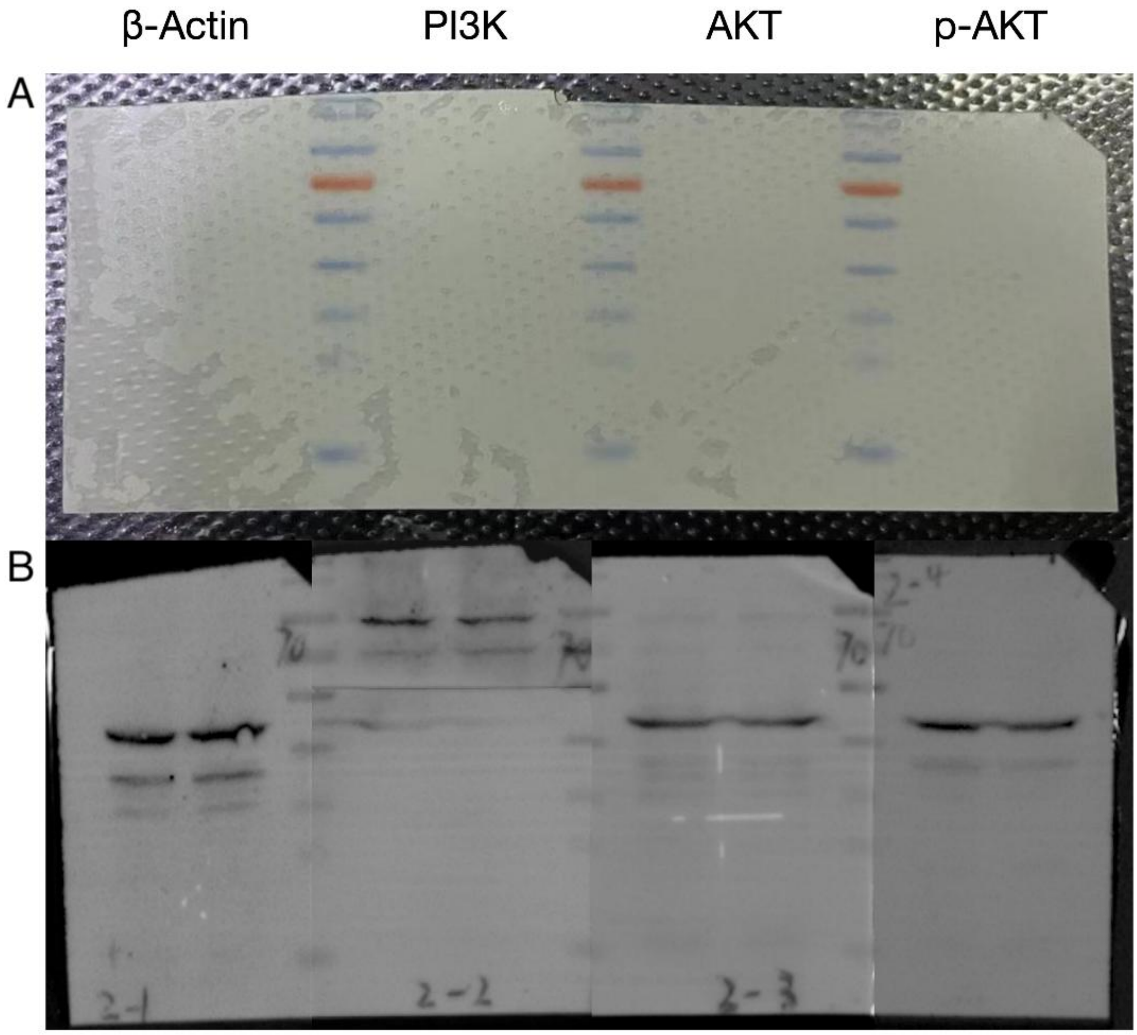

Supplementary Figure 1. PI3K/AKT the full uncropped gel and blot images (s) A. Original image of uncropped film; B. Image after hybridisation of PI3K/AKT/p-AKT antibody

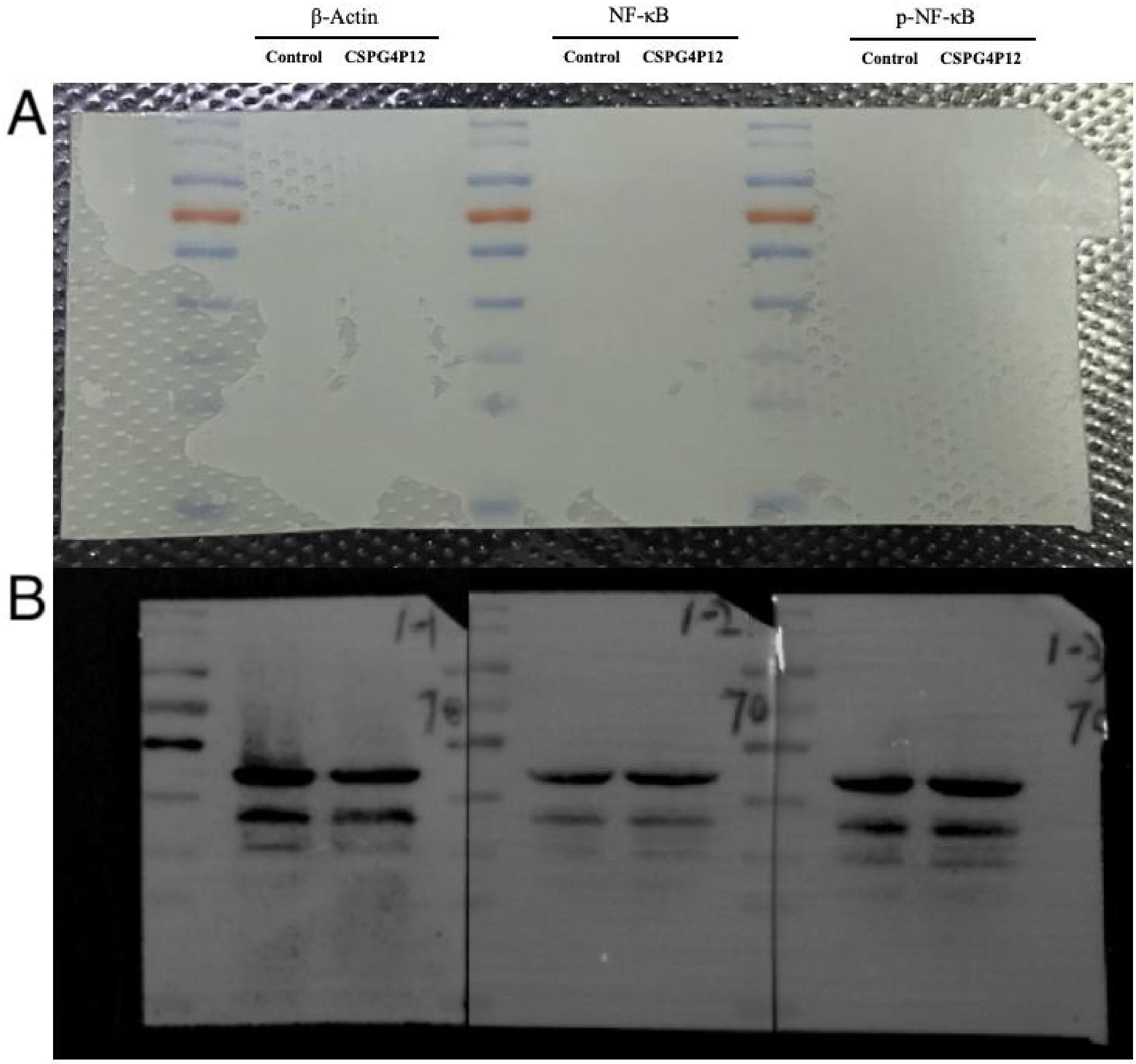

Supplementary Figure 2. NF-κB the full uncropped gel and blot images (s) A. Original image of uncropped film; B. Image after hybridisation of NF-κB/p-NF-κB antibody

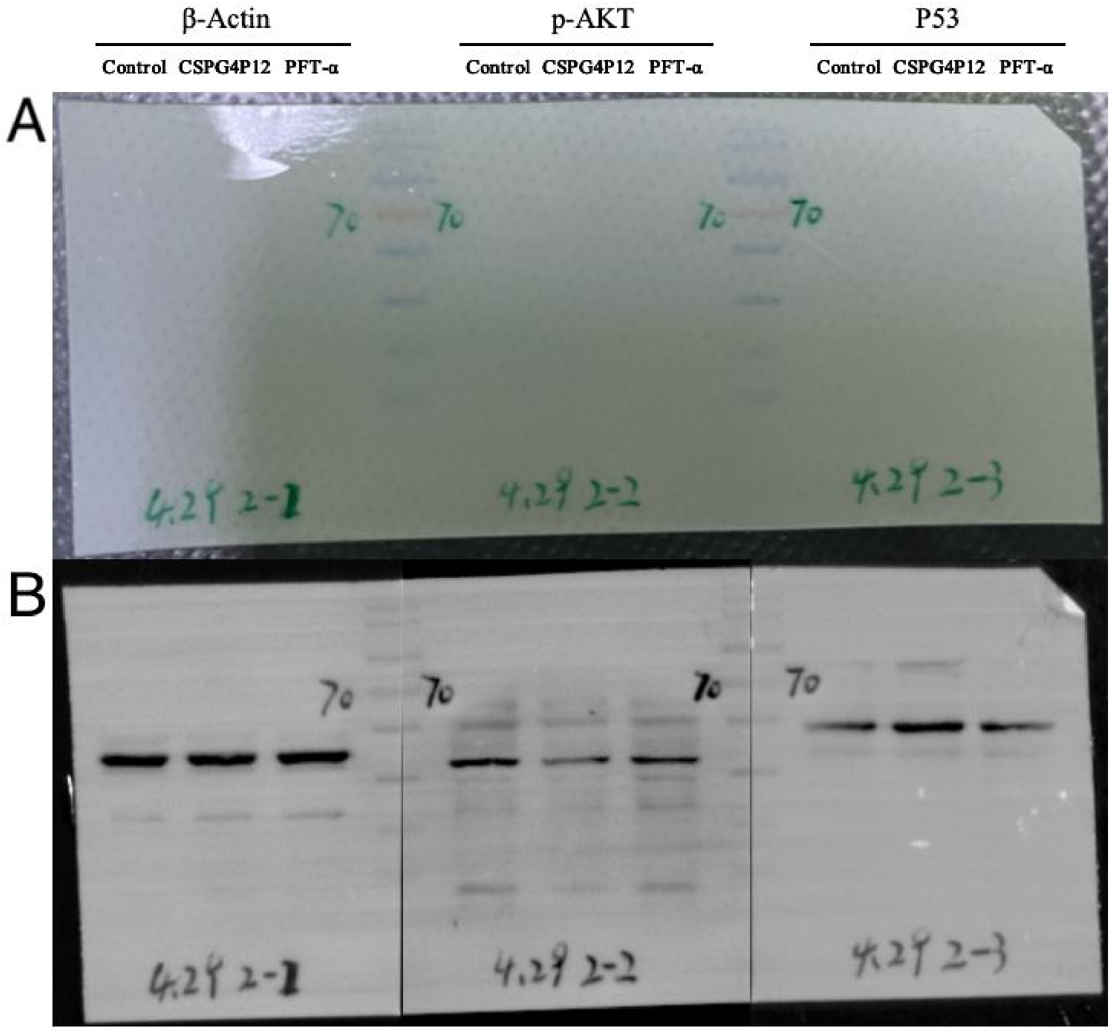

Supplementary Figure 3. P53/p-AKT the full uncropped gel and blot images (s).

A. Original image of uncropped film; B. Image after hybridisation of P53/p-AKT antibody
